# Reply to “Automated external defibrillator cabinets without scissors: a hidden barrier to effective resuscitation”

**DOI:** 10.1016/j.resplu.2026.101350

**Published:** 2026-05-05

**Authors:** Kentaro Omatsu, Gen Toyama, Eiji Hori, Yutaka Takei

**Affiliations:** a Department of Emergency Medical Sciences, Faculty of Medical Technology, Niigata University of Health and Welfare, Niigata, Japan; b Graduate School of Health and Welfare, Niigata University of Health and Welfare, Niigata, Japan

Dear Editor,

We thank Fijačko et al.^1^ for their interest in our study^2^ and for highlighting the practical issue of scissors availability in public automated external defibrillator (AED) cabinets. We also appreciate their perspective on the real-world variability in AED equipment.

We would like to clarify the interpretation of our findings, as this point is central to our discussion. Fijačko et al. state that we “demonstrated that standard stainless steel trauma scissors can efficiently remove clothing.” This does not accurately reflect the results. In our randomized controlled simulation trial involving 40 undergraduate students, the use of scissors for clothing removal was associated with a longer time from AED power-on to shock delivery. The median time was 118 s in the scissors group compared with 91.5 s in the no-scissors group, a statistically significant difference of 24 s (95% CI 6–39 s; *p* = 0.004). Pad placement accuracy did not differ between the groups for either the anterior (OR 1.00, 95% CI 0.13–7.89) or lateral pad (OR 0.67, 95% CI 0.19–2.33). Our conclusion explicitly states that these findings “do not support routine scissor use under the simulated conditions.”

The scissors used in our study were dedicated stainless steel trauma scissors (SÖHNGEN® Rescue Scissors), which Fijačko et al. described as relatively expensive and purpose-built for efficient cutting. Notably, defibrillation was delayed even with the use of these scissors. In this context, the real-world scenario they described, where scissors may be absent or of lower quality, highlights an important practical consideration and may further emphasize the challenges associated with their use by lay rescuers.

We share their concern regarding the lack of standardization in AED cabinet contents across countries, and we recognize that accessories such as razors, gloves, and face shield masks may contribute to safe resuscitation. However, scissors should not be considered a straightforward addition to public AED kits without considering the training required for their effective use. Our data suggest that, for untrained lay rescuers, the demands of scissors use may impede the primary goal of rapid defibrillation. Whether trained rescuers would show different outcomes remains an important question, and we believe that scissors may play a role when used by those with appropriate preparation.

Accurate interpretation of existing evidence, together with consideration of real-world implementation, will be essential as the community works toward optimizing AED preparedness for lay rescuers globally.

## Data sharing

The data supporting the findings of this study are available from the corresponding author upon reasonable requests.

## Declaration of generative AI and AI-assisted technologies in the writing process

During the preparation of this work, the authors used Claude for English language editing and stylistic refinement. After using this tool, the authors reviewed and edited the content as needed and took full responsibility for the content of the publication.

## CRediT authorship contribution statement

**Kentaro Omatsu:** Writing – review & editing, Writing – original draft, Conceptualization. **Gen Toyama:** Writing – review & editing. **Eiji Hori:** Writing – review & editing. **Yutaka Takei:** Writing – review & editing, Supervision.

## Declaration of competing interest

The authors declare no conflicts of interest.
